# United States College of Veterinary Surgeons

**Published:** 1896-05

**Authors:** 


### UNITED STATES COLLEGE OF VETERINARY SURGEONS.

THE closing exercises were held Thursday, April 16th, in the college lecture-hall, 222 C. Street, N. W., Washington, D. C. The diplomas were presented by Prof. C. B. Robinson to the following: Harry W. Reed, Red Bank, N. J.; and Andrew C. Seacord, Stamford, N. Y.

Prof. Robinson addressed the graduates, giving them much good advice, and Prof. S. D. Lamb also delivered an instructive address, carrying the students from their matriculation to graduation, and pointing out the rapid advances made in veterinary science in the past few years. He mentioned the important positions which the veterinarians were called to fill, and their duties as sanitary officers for the protection of the public, showing the close relation existing between animals and man, and closed by thanking the students for their kind attention during the session and wishing them godspeed in the humane work which they have undertaken.

The trustees announced that the college course would hereafter consist of three sessions of six months each, and thanked the gentlemen who had contributed to the support of the college in the past. During the session the faculty has been strengthened by the addition of Dr. J. P. Turner, U. S. A., and Dr. Walter W. Alleger, of Howard University.

